# Influence of Household Water Filters on Bacteria Growth and Trace Metals in Tap Water of Doha, Qatar

**DOI:** 10.1038/s41598-018-26529-8

**Published:** 2018-05-29

**Authors:** Jerome Nriagu, Chuanwu Xi, Azhar Siddique, Annette Vincent, Basem Shomar

**Affiliations:** 1 Department of Environmental Health Sciences, School of Public Health, University of Michigan, Ann Arbor, MI 48109 USA; 2 Unit for Ain-Zibaida and Groundwater Rehabilitation, King Abdulaziz University, Jeddah, Saudi Arabia; 3 Biological Sciences Program, Carnegie Mellon University in Qatar, Qatar, Saudi Arabia; 4 Qatar Environmental & Energy Research Institute, Hamad bin Khalifa University, Doha, Qatar, Saudi Arabia

## Abstract

Deteriorating water quality from aging infrastructure, growing threat of pollution from industrialization and urbanization, and increasing awareness about waterborne diseases are among the factors driving the surge in worldwide use of point-of-entry (POE) and point-of-use (POU) filters. Any adverse influence of such consumer point-of-use systems on quality of water at the tap remains poorly understood, however. We determined the chemical and microbiological changes in municipal water from the point of entry into the household plumbing system until it leaves from the tap in houses equipped with filters. We show that POE/POU devices can induce significant deterioration of the quality of tap water by functioning as traps and reservoirs for sludge, scale, rust, algae or slime deposits which promote microbial growth and biofilm formation in the household water distribution system. With changes in water pressure and physical or chemical disturbance of the plumbing system, the microorganisms and contaminants may be flushed into the tap water. Such changes in quality of household water carry a potential health risk which calls for some introspection in widespread deployment of POE/POU filters in water distribution systems.

## Introduction

Point-of-entry (POE) and point-of-use (POU) filters are becoming increasingly popular with consumers as a countermeasure for removing waterborne microbes and undesirable chemicals from water supply where there is no other treatment^1–3^. These mechanical filtration devices are now an indispensable component of desalinated water distribution systems in many countries where they are used to provide protection against intermittent changes in organoleptic properties of tap water^3,4^. These systems are also increasingly being deployed as an additional safety barrier in emergency cases where the quality of water from large scale (municipal) treatment and distribution systems are in doubt^5–7^. An endearing feature of note is that the units are relatively easy to use on existing plumbing infrastructure - they can simply be fitted to a single faucet to purify the portion of incoming water that is being used for drinking and cooking purposes (point-of-use), or to treat all the water coming into a house or facility at the point of entry.

Although POU/POE treatment devices can contribute to increased water safety and enhancement of water quality, they can have a number of undesirable attributes^6^. They can accumulate sludge, scale, rust, algae or slime deposits in the water distribution systems and potentially represent a temporary reservoir of undesirable contaminants^7,8^. Particulates in water distribution systems are invariably enriched in metals derived directly from corrosion of metallic pipes, joints and fixtures or indirectly through adsorption of dissolved metals by particles being transported, hence are a potential health threat. Filter membranes and adjacent interior surface of faucets can also serve as substrate for accumulation, growth and proliferation of pathogenic and non-pathogenic microorganisms^9–11^. It only takes a few microbial colonies trapped in the POE/POU devices to develop into a biofilm able to produce extracellular polymeric substances (EPS) even in oligotrophic environments^12^. Since bacterial surfaces and EPS components are negatively charged^13^, they tend to accentuate the capacity of POU’s to absorb positively-charged metal cations^14,15^, leading to immobilization of toxic metals in the POU devices^16^. Biofilms in water distribution systems can have a great impact on public health, especially when they harbor pathogens^17,18^. Unless the POU devices are maintained and replaced on a regular basis, they can quickly become incubators of microorganisms and concentrators of toxic metals^19^, which may periodically be discharged through the tap water. There is currently little information on the influence of POE/POU barriers on chemical and biological properties of household water from municipal water distribution systems.

Thermal desalination of brackish water accounts for over 99% of the drinking water in Qatar^20,21^. In general, water produced by desalination is low in minerals and total organic carbon (TOC) content and usually is aggressive towards materials used in the distribution pipes, storage and plumbing^22,23^. Drinking water in Doha remains highly demineralized despite efforts to mitigate its corrosivity with a variety of post-treatment measures including stabilization through addition of lime, carbonate alkalinity, corrosion inhibitors, and mixing with some source water. The household water distribution system in Doha can be regarded as a unique (synthetic) oligotrophic ecosystem with chemical and biological characteristics that influence the organoleptic properties of the treated water in ways that require the use of POU/POE filters by most households. This study was designed to characterize the chemical and microbiological changes in municipal water from the point of entry into the household plumbing system until it leaves from the tap in houses equipped with filters. Specific goals of the project include to (i) determine the effects of POE and POU systems on concentrations and types of trace metals in tap water; (ii) evaluate the influence of POE and POU on community structure of microbes in tap water; and (iii) assesses the association between the accumulation of trace metals and microbial growth in POE/POU filters in Doha where such devices are used in most of the households^24^. Although brands of POE/POU systems may differ, most mechanical filters work on the same basic principle, and we expect the effects observed in this study to obtain in other places as well. The results of the study should therefore contribute to our understanding of the risks and benefits of the growing worldwide use POE/POU systems by the public to enhance the safety of its tap water.

**Figure 1:**
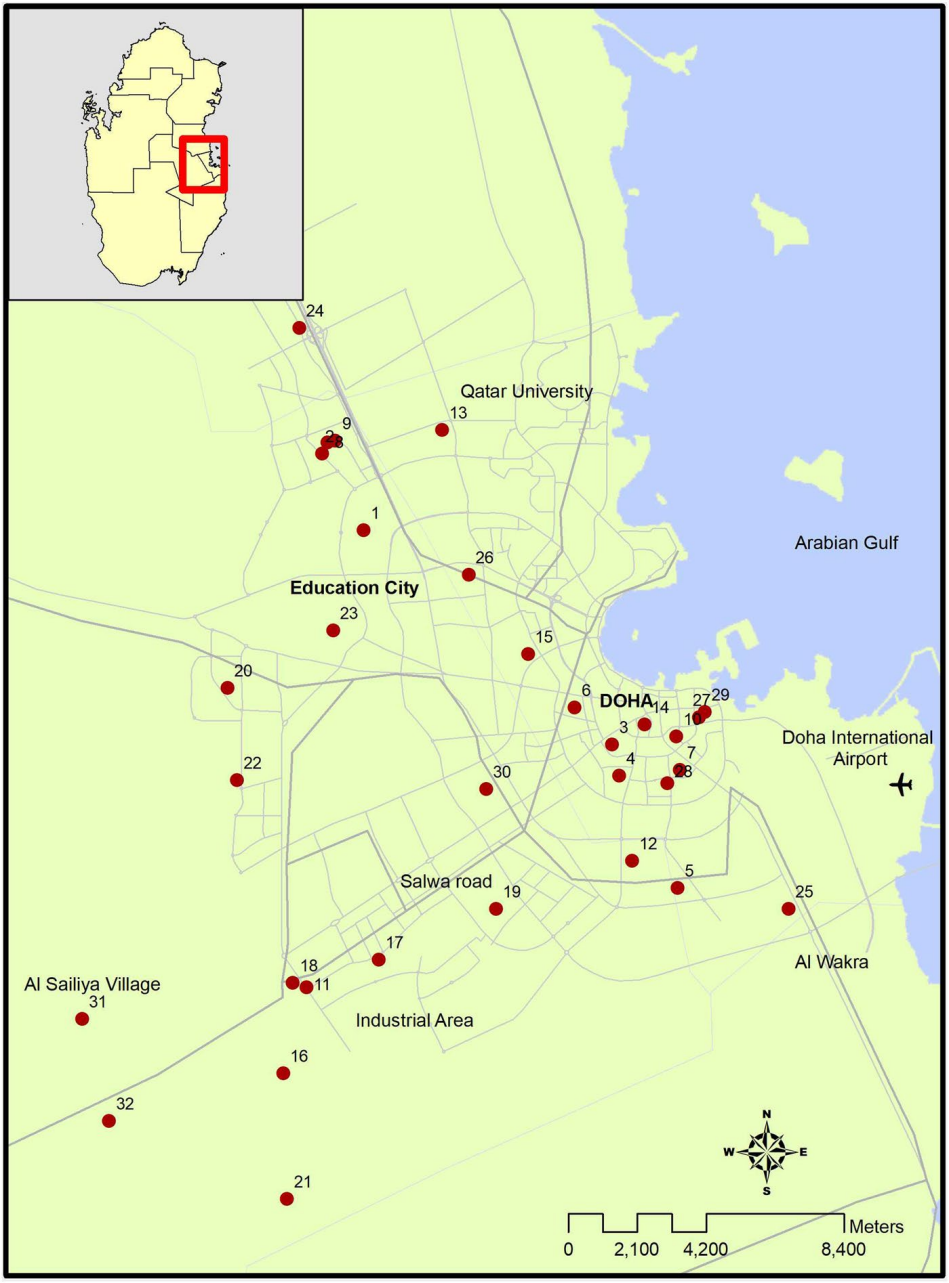
Locations of housing units in Doha that participated in the study. (Created using ArcGIS -a GIS software).

The study was carried out in the urban areas of Doha which has a population of around 1.5 million^21^. Convenience samples of 32 housing units were selected across the city of Doha (Fig. 1; Suppl. 1). The samples were collected in different zones of the city and at varying distances from the coast where the desalination plants are concentrated. The housing structures studied ranged from single family homes (~30%) to apartment/condominium units (~70%). Water samples were collected between September 2015 and August 2016 from the following four locations in the household supply line of each housing unit:

(i) Sample “A” – before the POE filter and should reflect the service line water (SLW)
(ii) Sample “B” – before the POU device which should reflect the water in household plumbing system or household water (HHW)
(iii) Sample “C” – tap water after the POU device or filtered tap water (FTW)
(iv) Sample “D” – water sample retrieved from inside the POU unit (or WIPD)

In addition, POU units retrieved from a subset of 10 houses were broken open and the residue trapped in each unit was retrieved for analysis. These 10 units were designed to represent different configurations (table top and under the sink varieties) and household use (single and multiple family dwellings).

Since there was little information on filter use in Qatar, a purposive survey instrument was used to gather information on maintenance and care of the filters, how often they were being changed (or cartridges replaced), customer education that participants had received or seen about their filters, the nationality of the respondent and the number of people in the household. The information was collected from adult members of participating households. In addition, the project staff took note of the specific type and model of each filter, where the filter was installed (table top or under the sink), label information about the cartridge and filter bed, any manufacturer certification on the filter unit, any performance indication device (PID) and how it would warn users, the type and location of the housing unit. Data collection was anonymized – participants did not provide their names, addresses, phone numbers or any unique identifying information.

## Results

**Figure 2:**
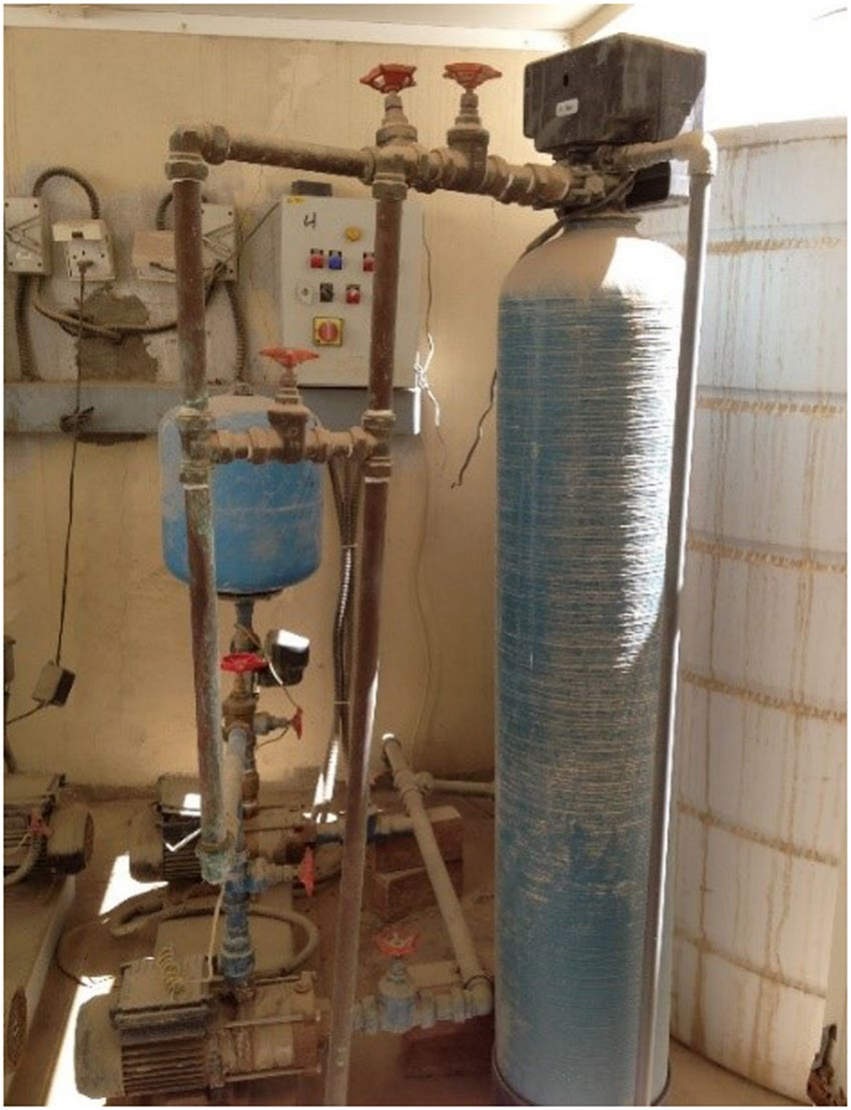
Typical point-of-entry filtering system (Lavender Village residence), Doha.

The survey and field observations^24^ showed that in most housing units in Doha, desalinated water from the service line is pumped into ground-level storage tank(s). These storage tanks typically have a capacity of 200–250 US gallons. Some buildings may have additional roof-top storage tank(s) to aid the water pressure in the home. The ground-level storage tanks often are equipped with point-of-entry (POE) filters intended to remove the dirt and debris that may be brought along by water in the municipal supply line (Fig. 2). This means that such POE systems are installed on service lines where the municipal water first enters a housing unit (typically referred to as villa in Qatar). They provide whole-house water filtration and the treated water is subsequently delivered to all taps, baths, showers, dishwashers, refrigerators, washing machines and toilets in the home. The POEs and associated storage tanks are usually located in an enclosed structure (shed) outside the building and hence are exposed to vagaries in the local temperature. A large number of villas in Doha were found to use the Pentair Master Filtration system which consists of layers of gravel, course silica sand, medium silica sand, fine silica sand and zeolite topped by a bed of activate carbon. They are capable of removing the different types of contaminants from treated water including pesticides, herbicides and insecticides; industrial contaminants; chlorine and disinfection byproducts; suspended solids; some heavy metals; and bacteria. Water from the POE is stored in a large outside tank from which it is distributed through the building. The POE systems provide high capacity filtration and can treat hundreds of gallons of water per day. The normal size of a mineral tank system for villas is up to 48 inches or more in height (see Fig. 2). Since whole-house POE filters are designed to produce general purpose water, most villas in Doha were found to be equipped with a combination of POE and POU systems in the effort to provide consumers with high quality water^24^. We were not able to retrieve and break open any of these large POE units.

**Figure 3:**
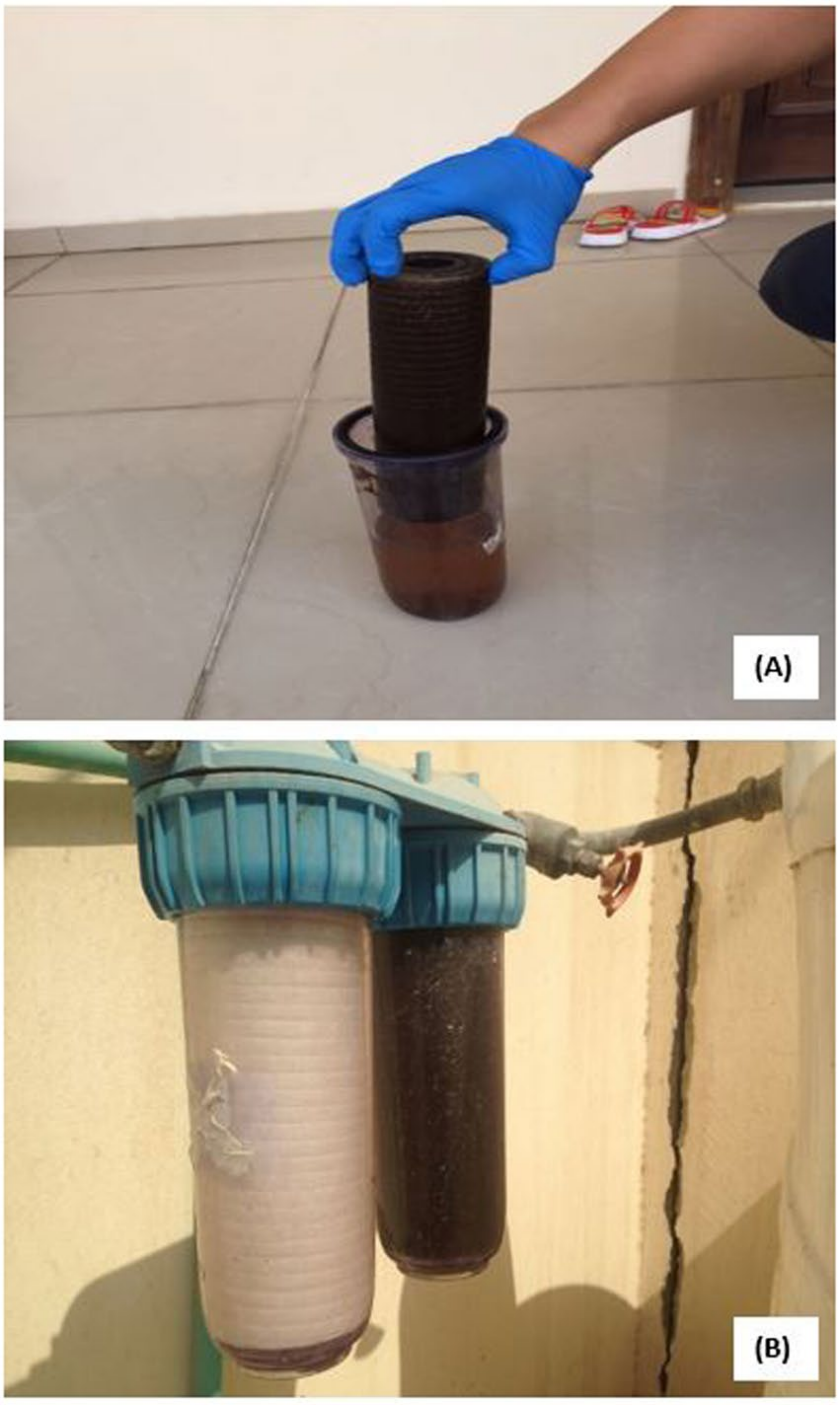
(**A**) Filter membrane showing heavy accumulation of residues; the beaker shows the brownish slurry within the POU unit. (**B**) Comparison of new filter (left) and one that had been installed for four months (right). The cartridge taken out of the filter is shown separately on the right.

Point-of-use (POU) devices were found to be an integral part of the tap water system in the kitchen of most (~80%) of the homes in Doha^24^. Most of the POUs are countertop types (Fig. 3A) although a few are mounted under the kitchen sink (Fig. 3B). Two main types of POUs were encountered in the Doha houses (i) granular activated carbon (GAC) based filters and (ii) polypropylene filters (PP). The PP filters can be further sub-divided according to their physical construction (i.e., hard surface, threaded etc.) and pore size (1 micron to 10 micron). The diversity of the POU devices was an interesting discovery in the study which raised the issue of how effective some of them were in removing any given contaminants from tap water^24^. The most common type of table-top units in Doha are different models of the activated carbon filtration system made by Panasonic and other companies^24^. They are designed primarily to reduce rust, mold and sediments (reduce turbidity) and help in removing color, taste and odor from water. According to specifications on the label, they are not able to remove dissolved inorganic compounds (including toxic metals) from water and no claims were made on their ability to remove parasites, radon, pesticides and herbicides or microorganisms.

During the project, the staff examined the POE/POU systems to see if they were equipped with any performance indication device (PID) and how it would warn users. None of the systems examined had any performance indication device – to show, for instance, when a cartridge was saturated and needed to be replaced. From a survey of participants^24^, it did not appear that there was a maintenance schedule for either the POE or POU systems^24^. According to the respondents, a new POU was installed when a new tenant moves into a flat or when a tenant complains about his/her filter unit. There was no plan in place to change the POU systems at regular intervals in many cases. Few people could remember when the POEs in the villas were last changed, or were subjected to back flushing. There was no plan in place to inform the consumers about the care and maintenance of their POU and POE systems^24^.

### Effects on Chemical Quality of Household Water

A number of previous studies on quality of municipal water supply in Doha had concluded that the tap water in the city is good for drinking purposes^25–27^. The results of this study affirm such conclusion in the sense that none of the water quality parameters (chemical or biological) that was monitored in this study exceeded its WHO’s guideline for drinking water^28^. However, our data suggest that there are characteristics of the distribution system which can render the water objectionable to many consumers. This study suggests that the offending properties are limited primarily to the household water distribution system and are associated with the POE/POU devices.

Figure 3B shows that the residues build up quickly on the filter membrane, hence are formed in large quantities in the water distribution system. The material captured by the POU unit (Fig. 3A) consisted of a slimy mass of brownish aggregate of sludge, scale, rust and organic matter. The slurry within the POU units (Fig. 3A) suggests that the residue cake on the filter membrane can be released and hence flushed into the tap water whenever there are significant physical disturbances or fluctuations in household water pressure. The number of people that had been turned off by seeing such sediment-rich colored water coming out of their tap is impossible to estimate but there were many anecdotal reports (in local newspapers and study participants) that this might be a frequent occurrence^24^.

Water in service lines of Doha tends to be soft (total dissolved solids ranged from 67–75 mg/L), mildly alkaline (pH of 7.5 to 7.6), is under-saturated with oxygen (total dissolved oxygen in the range of 1–6.8 mg/L) and can reach temperatures of 35–42 °C (Table 1). One can therefore infer that despite the post-treatment of the water after desalination, the city’s water still retains a corrosive character to the distribution line. The level of corrosion in the distribution system can be gleaned from the accumulation of corrosion products on the POE filters and in the storage tanks which can function as de factor traps. Visual examination of POE filter cartridges and membranes (see Fig. 3A,B) suggests that significant quantities of suspended materials are reaching the household water system from the main distribution line. The participants’ report that whole-villa filters are rarely replaced may explain the accumulation of sediments in storage tanks since the retention capacities of some of the filters are probably all spent. It is not possible to estimate the relative production of corrosion products within the household plumbing system using the current data, however.

**Table 1:** Major physico-chemical properties of household water in Doha.

*Parameter* ^*^	*Mean* ± SE^**^	*Range* ^**^	*Guideline Value* ^***^
HPC (A), CFU/mL	3.5 ± 2.5	1.0–6.0	100
HPC (B), CFU/mL	1218 ± 258	BDL − 5160	
HPC (C), CFU/ml	1229 ± 274	BDL – 5790	
pH (A)	7.6 ± 0.1	7.3–7.7	6.5–8.5
pH (B)	8.0 ± 0.1	6.1–8.4	
pH (C)	8.0 ± 0.1	7.5–8.4	
DO (A), mg/L	3.1 ± 1.9	0.7–6.8	
DO (B), mg/L	5.7 ± 0.3	2.9–7.5	
DO (C), mg/L	5.9 ± 0.3	3.5–7.5	
EC (A), µS/cm	142 ± 5.6	131–149	150–500 µS/cm
EC (B), µS/cm	172 ± 9.4	126–344	
EC (C), µS/cm	161 ± 9.9	89–338	
Temp (A), °C	37.9 ± 1.8	35.4–41.5	
Temp (B), °C	26.2 ± 0.8	19–35.1	
Temp (C), °C	25.3 ± 1.1	10.6–37.7	
TDS (A), mg/L	70.8 ± 2.3	64.7–75.1	110–250
TDS (B), mg/L	82.2 ± 5.2	6.0–172	
TDS (C), mg/L	80.4 ± 4.9	43–169	
TOC (B), mg/L	1.9 ± 0.4	0.8–9.2	4.0
TOC (C), mg/L	1.3 ± 0.1	0.4–3.2	
TOC (D), mg/L	2.7 ± 1.1	0.9–16.3	
TC (A), mg/L	17.8 ± 0.8	15.3–19	
TC (B), mg/L	17.7 ± 0.4	14.3–24.6	
TC (C), mg/L	16.9 ± 0.3	14.4–20.9	
TC (D), mg/L	16.9 ± 0.4	15.4–20.1	

^*^A = Service line water; B = Household water; C = Filtered tap water; D = Water in point-of-use Device; HPC = Heterotrophic plate count; DO = Dissolved oxygen; EC = Electrical conductivity; TDS = Total dissolved solids; TOC = Total organic carbon; TC = Total carbon.

^**^SE = Standard error of the mean; BDL = below detection limit.

^***^KAHRAMAA Requirements for Water Quality in Distribution System in Qatar [REVISED]; a blank value means that no guideline has been established for particular parameter.

What is not in doubt, however, is that a number of important measures of the quality of the tap water are distinctly different from those of samples from municipal main line and household plumbing systems (Tables 1–3). We found higher conductivity (EC), higher pH, more dissolved oxygen, higher total dissolved solids (TDS) but lower temperatures in filtered tap water (FTW) compared to the service line water (SLW) (Table 1). Bivariate regression models show that the pH of tap water was positively associated with conductivity (*r* = 0.516; *p* = 0.004) and total dissolved solids (*r* = 0.524; *p* = 0.004) (Table 4). The inverse relationship of pH with total carbon (*r* = −0.615; *r* = <0.001) (Table 4) points to significant buffering action by carbonate system. Since the electrical conductivity and total dissolved solids vary collinearly (*r* > 0.9; *p* < 0.0001), the *r*-values for the associations of these parameters with pH are very similar. The inverse relationship between hydrogen ion (H^+^) concentrations and TDS and EC is consistent with exchange reactions on the filter which may account for the increased concentrations of other cations in the tap water (see below

**Table 2:** Concentrations of major cations and anions (in mg/L) in household water of Doha.

*Water Quality Parameter* ^*^	*Mean* ± SE^**^	*Range*	*Guideline Value* ^***^
Cl (A)	61.2 ± 45.6	15.7–107	<80
Cl (B)	10.1 ± 1.0	5.0–27.9	
Cl (C)	9.0 ± 0.2	6.8–11.5	
Cl (D)	6.1 ± 0.4	3.7–12.8	
NO_3_ (A)	1.6 ± 0.9	0.7–2.4	10
NO_3_ (B)	0.6 ± 0.1	0.3–2.0	
NO_3_ (C)	0.5 ± 0.1	0.3–0.5	
NO_3_ (D)	0.4 ± 0.1	0.2–0.7	
SO_4_ (B)	1.4 ± 0.2	0.8–2.7	50
SO_4_ (C)	1.3 ± 0.1	1.0–1.6	
SO_4_ (D)	1.5 ± 0.4	0.3–10.5	
PO_4_ (A)	1.1 ± 0.1	0.8–1.1	0.01
PO_4_ (B)	1.3 ± 0.1	0.5–2.4	
PO_4_ (C)	0.8 ± 0.1	0.4–0.9	
PO_4_ (D)	0.9 ± 0.2	0.6–1.2	
Na (B)	5.3 ± 0.4	2.0–13.0	80
Na (C)	6.0 ± 0.5	0.9–13.2	
Na (D)	3.8 ± 0.2	2.0–6.0	
Mg (A)	0.9 ± 0.1	0.7–1.1	30
Mg (B)	1.3 ± 0.1	0.5–3.0	
Mg (C)	1.4 ± 0.2	0.5–3.8	
Mg (D)	1.0 ± 0.1	0.3–2.4	
Ca (A)	48.8 ± 19.3	25.1–87.1	80
Ca (B)	25.1 ± 0.6	18.1–34.7	
Ca (C)	25.7 ± 0.7	20.3–32.8	
Ca (D)	22.5 ± 1.3	12.5–37.3	
K (A)	2.4 ± 1.0	0.5–3.7	4.0
K (B)	0.4 ± 0.1	0.1–0.8	
K (C)	1.2 ± 0.4	0.1–8.6	
K (D)	0.4 ± 0.1	0.2–0.6	

^*^A = Service line water; B = Household water; C = Filtered tap water; D = Water in point-of-use Device.

^**^SE = Standard error of the mean.

^***^KAHRAMAA Requirements for Water Quality in Distribution System in Qatar^25^.

**Table 3:** Concentrations of trace elements in household water samples in Doha.

*Element* ^*^	*Mean* ± SE^**^	*Range*	*WHO Guideline (µg/L)*
As (B), ng/L	57 ± 6.6	BDL – 97	10
As (C), ng/L	42 ± 6.2	BDL – 90	
As (D), ng/L	47 ± 5.8	BDL – 94	
Cd (A), ng/L	30 ± 5.9	18–37	3
Cd (B), ng/L	25 ± 3.4	11–88	
Cd (C), ng/L	0.3 ± 0.1	BDL – 2.8	
Cd (D), ng/L	28 ± 6.7	BDL – 91	
Cr (A), µg/L	0.5 ± 0.1	0.3–0.7	50
Cr (B), µg/L	0.3 ± 0.1	0.1–0.8	
Cr (C), µg/L	0.3 ± 0.1	0.2–0.7	
Cr (D), µg/L	0.4 ± 0.1	0.1–0.8	
Cu (A), µg/L	43.7 ± 21	14.7–84.5	2000
Cu (B), µg/L	32.1 ± 4.2	6.8–93.5	
Cu (C), µg/L	27.8 ± 3.2	7.7–65.1	
Cu (D), µg/L	27.1 ± 4.4	2.3–84.1	
Fe (A), µg/L	19.3 ± 6.6	6.3–27.4	300^***^
Fe (B), µg/L	21.8 ± 1.9	8.4–51.2	
Fe (C), µg/L	21.3 ± 2.5	7.9–60.6	
Fe (D), µg/L	17.5 ± 2.8	7.5–69.9	
Mn (A), µg/L	5.0 ± 0.9	4.1–6.0	400
Mn (B), µg/L	2.5 ± 0.3	0.1–7.4	
Mn (C), µg/L	2.3 ± 0.5	0.1–9.0	
Mn (D), µg/L	7.6 ± 3.0	BDL – 49.2	
Ni (A), µg/L	6.6 ± 4.2	2.4–10.8	70
Ni (B), µg/L	7.7 ± 1.3	1.7–22.9	
Ni (C), µg/L	7.1 ± 1.3	1.2–23.7	
Pb (A), µg/L	3.7 ± 2.1	1.4–5.9	10
Pb (B), µg/L	2.4 ± 0.6	0.1–9.4	
Pb (C), µg/L	0.3 ± 0.1	BDL – 2.4	
Pb (D), µg/L	0.5 ± 0.3	BDL – 5.2	
U (A), ng/L	28 ± 5.0	17–28	30
U (B), ng/L	23 ± 2.0	12–70	
U (C), ng/L	38 ± 5.0	11–90	
U (D), ng/L	33 ± 5.0	BDL – 76	
Zn (A), µg/L	21.8 ± 8.4	8.4–37.2	3000^***^
Zn (B), µg/L	18.0 ± 1.7	5.6–38.8	
Zn (C), µg/L	19.2 ± 1.9	4.5–45.2	
Zn (D), µg/L	21.1 ± 2.6	4.0–52.4	
B (B), µg/L	22.5 ± 2.4	9.7–58.9	
B (C), µg/L	20.2 ± 1.1	11.2–28.3	
B (D), µg/L	17.8 ± 1.2	11.5–23.8	
Al (B), µg/L	25.6 ± 3.5	5.4–62.1	200^***^
Al (C), µg/L	26.9 ± 3.9	6.3–65.3	
Al (D), µg/L	19.7 ± 4.9	4.9–64.7	

^*^A = Service line water; B = Household water; C = Filtered tap water; D = Water in point-of-use Device.

^**^SE = Standard error of the mean.

^***^KAHRAMAA Requirements for Water Quality in Distribution System in Qatar^25^.

**Table 4:** Association of bacterial counts with major physico-chemical properties of filtered tap water in Doha.

Parameter^#^		CFU/ml	pH	DO	Temp	EC	TDS	TOC	TC
CFU/ml	R	1	−0.052	0.163	−0.311	−0.139	−0.136	0.415	−0.098
	P		0.790	0.506	0.101	0.474	0.480	0.061	0.611
	N		29	19	29	29	29	21	29
pH	R		1	0.017	−0.350	0.518^**^	0.523^**^	0.049	−0.619^**^
	P			0.946	0.063	0.004	0.004	0.832	0.000
	N			19	29	29	29	21	29
DO	R			1	−0.802^**^	0.259	0.255	0.004	−0.034
	P				0.000	0.284	0.291	0.985	0.889
	N				19	19	19	19	19
Temp	R				1	−0.216	−0.222	−0.359	0.426^*^
	P					0.261	0.248	0.109	0.021
	N					29	29	21	29
EC	R					1	1.000^**^	−0.151	−0.434^*^
	P						0.000	0.514	0.019
	N						29	21	29
TDS	R						1	−0.150	−0.436^*^
	P							0.517	0.018
	N							21	29
TOC	R							1	0.031
	P								0.894
	N								21

^**^Correlation is significant at the 0.01 level (2-tailed).

^*^Correlation is significant at the 0.05 level (2-tailed).

^#^R = Pearson correlation coefficient; P = Significance level; N = Number of valid samples; CFU (HPC) = Heterotrophic plate count; DO = Dissolved oxygen; EC = Electrical conductivity; TDS = Total dissolved solids; TOC = Total organic carbon; TC = Total carbon.

Temperature was inversely and strongly correlated with dissolved oxygen (*r* = −0.802; *p* < 0.001) (Table 4), as to be expected. The stimulatory influence of temperature on biotic growth is indicated by positive correlation with total carbon in the samples (*r* = 0.426; *p* = 0.021). Temperature is an environmental factor that is well known to affect the growth of microorganisms in water distribution systems^8^. The optimum temperature for the microbial growth inside POU devices has been reported to be 30 °C, which falls within the common range (19–42 °C) for water samples in this study (Table 1). Besides temperature, total carbon is inversely associated with pH (*r* = −0.619; *p* < 0.001), electrical conductivity (*r* = −0.434; *p* = 0.019), and total dissolved solids (*r* = −0.436; *p* = 0.018) (Table 4). No association was found between total carbon and total dissolved carbon suggesting that inorganic carbonate species are the dominant forms in the samples.

The mean concentrations of major ions (Cl, NO_3_, PO_4_, SO_4_, Na, K, and Ca) were significantly higher in the service line water compared to those of the tap water (Table 3). The relatively high concentrations of these elements reflect the fact that amendment of desalinated water with local seawater is a common post-treatment procedure in Qatar^20^. Calcium in city water may also be related to the use of lime and/or other calcium-containing salts in the adjustment of the pH of desalinated water^3,28^. The results show that most of the essential minerals added to the water in post-desalination treatment have largely been removed by the POE and POU filters. The net effect is that the tap water was made more highly demineralized. Ingestion of such water has been suggested to be a risk for metabolic diseases and can potentiate some hallmarks for cancer^29–32^. Such likely consequence is contrary to the intended use of filters, which is to make the tap water safe.

The concentrations of the trace elements in the samples were highly variable (see the minimum and maximum values in Table 3), presumably reflecting the diversity in types of POE and POU devices in use in the city, the complex behavior of each element in the water distribution system, and the post-treatment processes. It is believed that the effort to stabilize and reduce the corrosivity of desalinated water using a variety of post‐treatment measures including addition of carbonate alkalinity, corrosion inhibitors, and mixing with source water (partially treated) or saline groundwater^20,23^ was an important source of some undesirable trace elements (such as U, As, B, and Pb) in tap water

Temperature of the household water (HHW) was positively associated with Cu (*r* = 0.507; *p* = 0.004), Cr (*r* = 0.524; *p* = 0.002), Fe (*r* = 0.376; *p* = 0.040), Ni (*r* = 0,576; *p* = 0.001). These associations likely reflect the influence of high ambient temperatures in Qatar on the corrosion of metallic conduits and fixtures by the soft desalinated water. Temperature of tap water likewise was positively associated with Cr (*r* = 0.601; *p* = 0.001), Cu (*r* = 0.436; *p* = 0.020), Fe (*r* = 0.447; *p* = 0.017), Ni (*r* = 0.422; *p* = 0.025). Since the average temperature of the HHW (26 ± 0.8 °C) is very similar to that of FTW (25 ± 0.8 °C), it is inferred that the inherited effects of corrosion on the concentrations of these metals are not overprinted by the retention of these metals in the POU systems (see above). We have no explanation for the inverse relationship between the temperature of tap water and Pb (*r* = −0.546; *p* = 0.009) as well as B (*r* = −0.551; *p* = 0.012). The association of temperature with Pb or B in HHW and SLW was not statistically significant, however.

### Effects on Microbial Growth and Biofilm Formation

Previous studies had reported that little or no regrowth of heterotrophic bacteria occurred if the assimilable organic carbon (AOC) concentration is less than 10–15 *μ*g C/L^32^. Since the average total organic carbon concentration in SLW was below the detection limit (Table 1), it is inferred that bacteria regrowth and biofilm formation were limited in the municipal main distribution and service line waters. Besides the low levels of organic carbon, microbial growth in SLW could also be limited by lack of minerals, nutrients, the presence of residual disinfectants, etc^33^. Our data demonstrate that the regrowth of bacteria in household water occurs primarily after entering individual housing unit. In particular, we found that there were accumulations of minerals and organic matter and essential trace elements on filters used in POE/POU devices, which were likely sufficient to promote bacteria growth and biofilm formation on their surfaces. Other factors that might have contributed to bacteria regrowth in HHW included low residual disinfectant, high temperature in storage tanks, and relative long residence time in storage tanks^34^.

**Figure 4:**
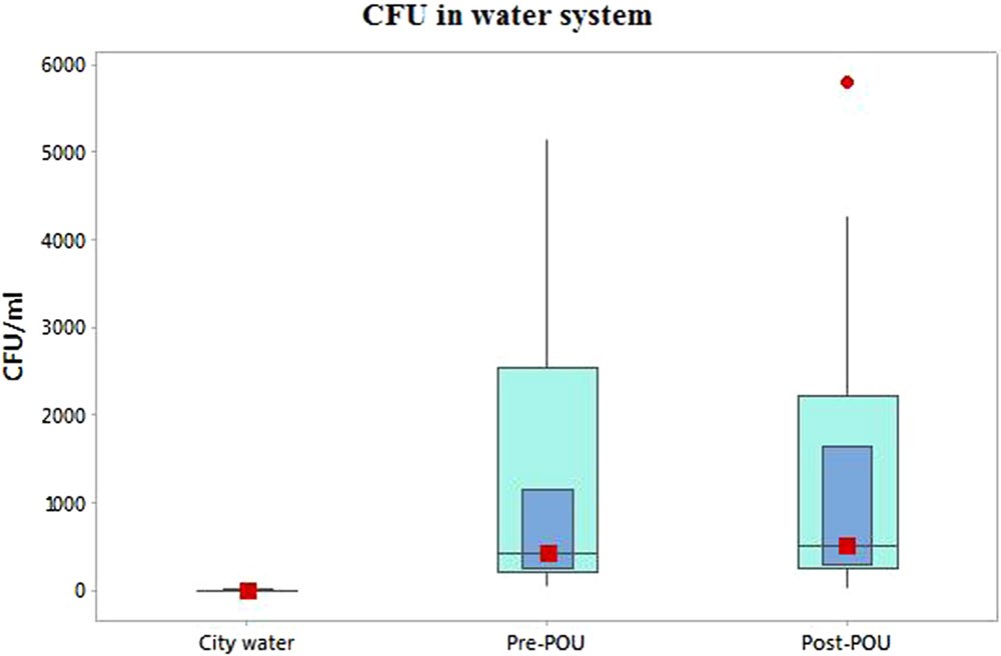
HPC counts in city (service line) water, household water (pre-POE water) and tap water (post-POU filter). (Created using Minitab® version 17.1.0 (2013), Minitab Inc.).

The big difference in TOC concentrations of water in the POC units compared to that of the HHW is consistent with the reports that trapped organic materials within the filter can promote the growth of heterotrophic bacteria and the formation of the biofilms within the filter devices^8,35^. The fact that there was no difference in the average concentrations of the CFUs in tap water and household water (Fig. 4) would seem consistent with the fact that POU systems are the center of active biofilm formation and bacteria growth. There were large differences in CFUs of HHW and tap water in individual households as shown in Fig. 4. The concentration of the total organic carbon (TOC) in tap water (1.3 ± 0.12 mg/L) relative to those of HHW (1.9 ± 0.39 mg/L) and water in the POU devices themselves (2.7 ± 1.1 mg/L) indicates that the filters are not removing all the organic materials. In other words, the POU devices act as leaky micro-ecosystems in spite of their ability to trap and retain organic matter. A study by Su *et al*.^8^ showed that the intensity of bacteria (biofilm) adhering on the surface of activated carbon filter inside a POU device was weak which allowed the bacteria to be washed out even at flow rate as low as 0.20 L/min. Our results are consistent with this observation. It has also been reported that carbon filter may be less effective in removing microbial contaminants from water compared to removal of organic compounds^1,8^ – a possible partial explanation for the differences observed in TOC concentrations.

Microorganisms that form biofilms have evolved various mechanisms to sequester and accumulate trace metals from water to ensure their survival^36,37^. Trace elements can affect microbial growth both as nutrients and toxins. We used multivariate regression models to explore the relationships between CFUs and the concentrations of trace elements in the water samples. No trace element was significantly associated with CFU in the tap water or household samples. This is not surprising since none of the toxic elements (As, B, Cd, Pb, U and Al) was found in concentrations (see Table 3) that approach values that are likely to be associated with adverse effects on microbial populations^38^. On the other hand, the concentrations of the essential elements (Cu, Fe, Mn and Zn) are somewhat elevated and hence do not seem to represent a nutritional stress to the biofilm colony. Linear bivariate models showed an inverse relationship between Cr and CFU (*r* = −0.414, *p* = 0.036) in tap. Little is currently known about the effects of Cr deficiency on biofilm formation or viability. Unsaturated biofilms exposed to Cr have been shown to result in in elevated extracellular carbohydrates, protein, DNA, and EPS sugars that were relatively enriched in *N*-acetyl-glucosamine, rhamnose, glucose, and mannose – which are important hallmarks in biofilm development^39^. Whether Cr deficiency can reverse these processes is conjectural at this point.

Although HPC bioassay has little value as an indicator of pathogens present in water, it can be used in assessing the integrity of distribution systems and potential presence of biofilms^25^. A huge difference in CFU was found between the city water (~4 counts per milliliter or CFU/mL) and both the household water (1212 CFU/mL) and the tap water (1229 CFU/mL) (Table 1). None of the macronutrients was associated with CFU either in the tap water or the service line samples (Table 2). The results of regression models to assess the influence of the principal physical and chemical properties of water (pH, temperature, DO, TOC, TC, and TDS) on CFU are shown in Table 4. There was a significant negative association between CFU and temperature of the SLW (*r* = −0.371, *p* = 0.040), indirectly implicating the effects of water tanks which are exposed to ambient temperatures. The CFU was weakly associated with TOC in tap water (*r* = 0.415, *p* = 0.061). With the exception of these two factors, CFU was not associated with the other physical-chemical parameters of the water samples from the sampling locations.

**Figure 5:**
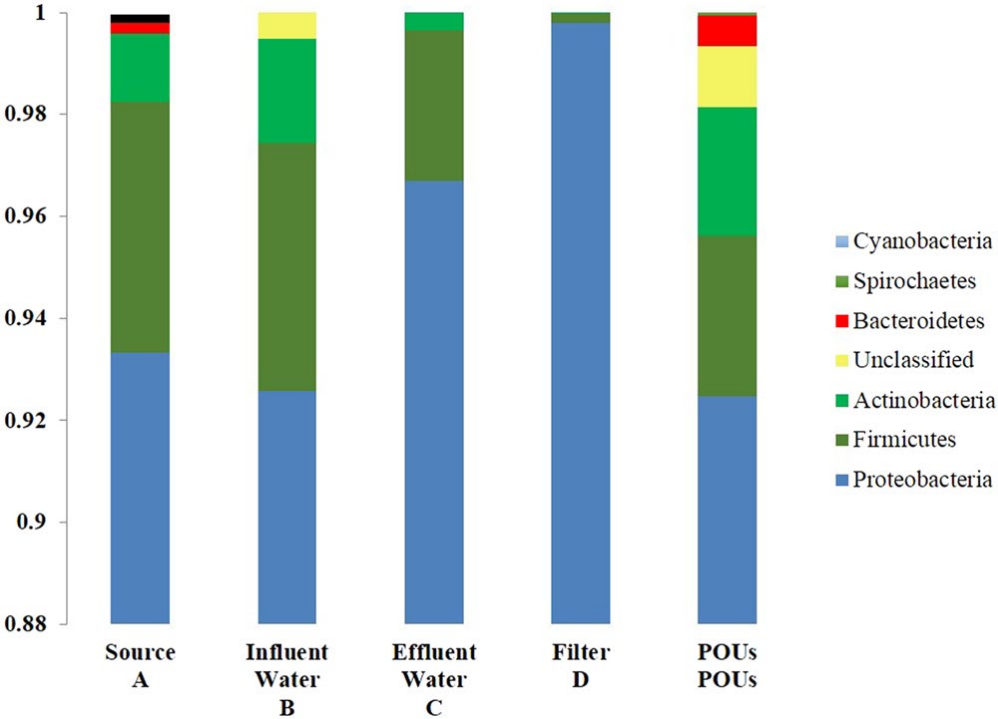
Community structure of microorganisms in different water samples. A = service line water; B = household water; C = tap water; D = water within the filter; POUs = residues on filter membranes. Y-axis shows the relative proportion of each group of microorganisms in the sample.

We examined the influence of POU devices on the microbial community structure using DNA sequencing techniques, and found that in spite of the very low HPC, the microbial populations in the service line waters were surprisingly quite diverse. This study identified seven taxonomically aligned strains in the HPC isolates with the proteobacteria accounting for over 90% of the microbes in each samples (Fig. 5). Studies of municipal water samples in other countries have likewise identified this strain to be most abundant in isolates in the distribution systems^32,40^. The DNA fingerprinting results show significant differences in diversity and relative abundance of HPC microbes in city water, household water and tap water (Fig. 5). In general, the same major strains (proteobacteria, fermicutes and actinobacteria) are found in comparable relative abundance in the service line and household water samples (Fig. 5). On the other hand, the community structure of the isolates from residues in the POU is quite different from that of tap water. This suggests that the POU system can reduce the microbial strains in the tap water probably by intercepting specific species and/or by rendering them non-culturable. The difference in community structure of the water and residues in the POU is equally remarkable (see Fig. 5) and suggests that other than proteobacteria, most of the HPC microbes in the POU systems were in attached biofilms. This study thus suggests that the POE/POU devices not only mediate the microbial regrowth but also can shape the community structure in the household water (Fig. 5).

As noted previously, nearly all the drinking water in Doha is derived from thermal desalination process at temperatures that would normally kill most of the microorganism. To safeguard against any bacterial contamination that may be introduced by the post-treatment process and in the water distribution system, however, small amounts of chlorine dioxide (ClO_2_) are added to the water system for disinfection purposes. A recent study has reported on the concentrations of ClO_2_ and its disinfection by-products (namely chlorite, chlorate, and trihalomethanes (THMs)) in a large number of water samples collected from desalination plants, reservoirs, and consumer outlets (mosques) in various locations in southern and northern Qatar^27^. The study found that the ClO_2_ concentrations in samples from desalination plants ranged from 380 to <20 *µ*g/L with the mean value being 170 *µ*g/L^27^. Concentrations of ClO_2_ in reservoirs were found to range from 240 to <20 *µ*g/L. These data for desalination plants and reservoirs point to inconsistent chlorination of the city’s drinking water. It was further reported that the mean residual ClO_2_ level at the mosques was <40 *µ*g/L implying that over 75% of the added disinfectant was being degraded during prolonged travel and stagnation of water in the distribution network^28^. Mean chlorite concentrations ranged from 13–440 *µ*g/L for desalination plants, 78–320 *µ*g/L for reservoirs and 85–440 *µ*g/L for mosques while the chlorate levels averaged 36–280 *µ*g/L in the different desalination plants, 11–200 *µ*g/L in the reservoirs and 11–150 *µ*g/L at the mosques^27^. A number of trihalomethanes were detected (including CHBr_3_, CHCl_3_, CHCl_2_Br, and CHClBr_2_) which ranged in concentration from 0 to 77 *µ*g/L and averaged 5 *µ*g/L^27^. The reported concentrations for the disinfection by-products were below the WHO^23^ guidelines and the operational limits set by Kharaama^25^. The low concentrations of ClO_2_ in relation to those of the disinfection by-products (DBPs) and the fact that tabletop POUs are designed to remove >95% of the residual chlorine are evidence that municipal water disinfection was having minimal effect on microbial growth and biofilm formation in the filter microcosms.

Residues on filter membranes can include inorganic compounds, colloidal and particulate matter, dissolved inorganic and organic compounds and various groups of microorganisms^40,41^. The concentrations of trace elements in residues from 10 POU filters are shown in Table 5. They consist mainly of compounds of copper (>2.8% dry weight), calcium (0.8% dry wt), manganese (0.9%), iron (0.6%), magnesium (>0.2%), and zinc (0.3%). Such metal-enriched compounds could result from the corrosion of metallic pipes, joints, fittings and control valves and the faucet. The dark coloration (Fig. 2) implies that the residues are rich in organic matter. The composition of this material formed in the synthetic environment of the water distribution ecosystem is unlike anything we have seen in the literature. The high concentration of lead (563 ± 682 *µ*g/g) is notable as it represents a significant hazard in the residues. The concentrations of other toxic metals/metalloids in the residues are relatively low including Ba (34 *µ*g/g), As (2 *µ*g/g), Cd (2.8 *µ*g/g), Co (14 *µ*g/g), Cr (9.3 *µ*g/g), and Se (3.8 *µ*g/g) (Table 5).

**Table 5:** Concentrations of trace and major elements in residues from point-of-use devices.

Metal/Metalloid	Mean ± SE^**^	Range^**^
Mg (mg/g)	1.8 ± 0.5	BDL – 4.5
Ca, mg/g	8.0 ± 2.4	1.8–22
As, µg/g	2.0 ± 0.6	BDL – 5.1
Ba, µg/g	34.4 ± 9.4	3.1–78.2
Cd, µg/g	2.8 ± 0.8	0.2–6.1
Co, µg/g	13.9 ± 4.1	0.4–37.3
Cr, µg/g	9.3 ± 3.9	0.1–31.6
Cu, mg/g	27.8 ± 7.5	0.2–50
Fe, mg/g	6.2 ± 2.6	BDL – 20.6
Mn, mg/g	9.1 ± 2.8	BDL – 20.1
Ni, mg/g	0.51 ± 0.15	0.1–1.1
Pb, µg/g	613 ± 244	7.8–2000
Se, µg/g	3.8 ± 1.2	0.7–13.7
U, µg/g	2.5 ± 0.8	BDL – 5.0
Zn, mg/g	2.9 ± 0.9	0.1–8.8
B mg/g	BDL	
Al mg/g	BDL	

^*^Number of samples = 10.

^**^SE = Standard error; BDL = Below the detection limit.

## Discussion

Our data show that the POU systems are exercising a strong influence on the microbiome in the household water distribution system. The HPU was <10 CFU/mL in all samples collected before the POE (service line water) (Table 1), and below the maximum permitted levels of 100 CFU/mL in distribution system (service line before the customer’s connection point) and 10 CFU/mL for water at desalination plants delivery point have been adopted by Kahramaa (the Qatar General Water and Electricity Corporation) responsible for drinking water supply in the country^25^. In other words, there was little evidence for microbial growth in the main distribution lines which can be considered a stressful environment where nutrient limitation, high temperatures and disinfection with chlorine dioxide limit the potential for microbial growth. By contrast, the HPC in household (post-POE) samples reached over 5000 CFU/mL and averaged 12-fold higher than the guidance value (Table 1), clearly indicating enhanced microbial growth and a significant deterioration of quality when the water gets into the household distribution system. The underlying reasons for the development of HPC in household water distribution system from the small initial populations in the city water are unknown. Possible contributing factors include the concentration of cells on growth-conducive filter membranes, changes in relative ratios of attached to unattached microbes leading to formation of biofilms, increase in food source from degradation of bacterial biomass (see TOC and TC in Table 1), and lowered temperature in the home environment.

Globally, desalination is promoted as a source of quality-controlled, premium form of potable water that is devoid of contaminants. It is sometimes regarded as green technology in the sense that it reduces the pressure to use freshwater resources and the tendency to disrupt the natural ecological balance^42^. Paradoxically, the general public in Qatar has remained skeptical about their water supply and most refuse to drink the tap water based primarily on perceived health risks. In fact, negative consumer perceptions about desalinated water remain prevalent across the Gulf Cooperation Council (GCC) countries and in many parts of the world^43–46^. Bottled water now represents the primary source of water for a large majority people in Qatar and other GCC countries^26^. Widespread adoption of point-of-use/point-of-entry water treatment devices is another strategy designed to allay the public’s concerns about local tap water^6,8^. While this study shows that the tap water in Doha meets all the national and international guidelines used in judging drinking water to be safe, it cannot eliminate the concerns about how “healthy” the water actually is. Current water quality guidelines are designed to prevent exposure to toxic amounts of contaminants (chemical or biological) in water. However, recent studies increasingly show that the exposure-response relationships for most essential minerals (minor and major trace elements) in water have hockey-stick profiles for sick patients and are U-shaped for healthy populations^47,48^. In other words, ingestion of demineralized water carries health risks, just like water with high concentrations of the major and minor elements. While the chemical data for tap water in Doha are well below the established thresholds (current guidelines) for toxicity, they sometimes fall below the low-end limits that have been associated with electrolyte imbalance and adverse health effects. Until we have a better understanding of the health effects of long-term ingestion of demineralized water, any claim that desalinated tap water is safe cannot rule out that it may not be quite so healthy. Coincidentally, the unintended consequence of the POE/POU filters is increased demineralization of the tap water.

In spite of the rich data from the project, the study has a number of limitations. In the first instance, the number of households (32) is small and might not have included all the types of POE and POU systems in use in the country. We do believe that the most common types that were in use in the city’s households were included in the study, however. In view of the large number of variables and likely confounders that have been assessed in the paper, the possibility of Types I and II errors in the reported associations cannot be completely ruled out with such sample size. Another limitation is that only total concentrations of metals/metalloids were determined in this study; no attempt was made to measure the chemical species present in the samples. The inter-element discussion should therefore be taken with caution considering that the forms of an element can determine its interactions with other elements in the POE/POU ecosystem. Culture-dependent technique was used in assessing microbial populations in the samples. This test can only detect a small proportion of the microorganisms that are present in the sample (limitation of low culturability), and the population recovered can differ according to the method and conditions applied^35^. In addition, the bioassay has little value in detecting pathogens present in samples or as an indicator of health risk associated with exposure to the water^32,34^. The HPC data from this study therefore cannot be used to make any safety claims about the tap water in Qatar.

## Conclusions

We found significant concentrations of Cu (27.4 ± 17.1 *µ*g/L), Fe (21.3 ± 13 *µ*g/L), Zn (19.2 ± 10 *µ*g/L), B (20.2 ± 5 *µ*g/L), and Al (26.9 ± 18 *µ*g/L) in tap water of Doha, but the levels of toxic metals of common concern, namely arsenic (<0.1 *µ*g/L), cadmium (<0.1 *µ*g/L) and lead (0.34 *µ*g/L) were low. Tap water in the city had higher conductivity, pH, total dissolved oxygen (DO), total dissolved solids (TDS), total organic carbon (TOC) but lower temperatures compared to water in the service lines. A huge difference in microbial plate count was found between the service line water (~4 CFU/mL) and tap water (1229 CFU/mL). Residues trapped by the filters consisted mainly of compounds of Cu (>2.8% dry weight), Ca (0.8% dry wt.), Mn (0.9%), Fe (0.6%), Mg (>0.2%), and Zn (0.3%). The residues also contained elevated levels of Pb (563 *µ*g/g average), which may be of concern if they are flushed into tap water.

This study shows that POE/POU devices can induce a deterioration of the quality of tap water in Doha. The low bacteria count (HPC) found in service line water (collected before the point-of-entry and storage tanks), implies that microbial regrowth in finished desalinated water is insignificant presumably because of very low concentrations of organic matters, minerals, nutrients and the presence of residual disinfectants. By contrast, the CFUs of household and tap water samples were about 12-fold higher that the values for service line water. Equally important, this study documents significant differences in the chemical properties of the tap water compared to the service line water. These results suggest that the installed POE and POU filters and storage tanks have changed the chemical and biological characteristics of household water compared to treated municipal water. The filters also promote microbial growth and biofilm formation in the household water distribution system. The observation that the quality of the water is not exactly maintained from the treatment plant to the consumer’s tap because of the mechanical filters should be a matter of some concern.

Visible sediments in storage tanks and non-sanitary effects of the filters are frequently mentioned as a factor in rejection of tap water by city residents. Analysis of the residues trapped by the filters found no enrichment of toxic metals other than lead. Although the residues may not be a health hazard, they portray a visible esthetic property that can influence the drinking water behavior of many people in Doha. An unintended consequence of the POE/POU systems is the removal of some of the essential minerals and residual disinfecting chlorine used in post-treatment from the water. This effect is likely to make the water less “healthful”^31,47,48^, although this is not usually the concern of the consumers.

## Methods

### Sample Collection and Field Measurements

The following samples were collected and treated at each location:

(i) 250 ml of water sample in properly cleaned high density linear polyethylene (HDLPE) bottle and approximately 0.5 mL of concentrated nitric acid was added as preservative. The acidified sample was put in a cooler box and taken to the laboratory where it was stored at 4 °C.
(ii) Un-acidified 250 ml of water sample in HDLPE bottle for anion/cation analysis by ion chromatography (IC). The sample was stored in a refrigerator prior to IC analysis.
(iii) 40 ml water sample in amber glass bottle for TOC analysis and preserved with sulfuric acid. The samples were submitted within two weeks of collection to the total organic carbon (TOC) analyzer laboratory at the College of North Atlantic in Qatar (CNA-Q) for analysis.

In addition to water samples, the POU filter cartridge was removed and transported to the laboratory in a cooler box.

At each sampling location, physical-chemical parameters (pH, electrical conductivity (EC), temperature, total dissolved solids (TDS), and dissolved oxygen (DO)) were measured in each sample on site with HANA HI9829 multi-meter (Woonsocket, RI). Geographical location of the site was recorded with the built-in GPS of the meter. Appropriate aliquots of each water sample were collected for metals (total and dissolved), non-metals, total organic carbon (TOC) and heterotrophic plate count. The samples were kept in a cooler box and transported to laboratory freezer.

### Laboratory analysis

Once returned to the lab, the cartridge was opened and the contents apportioned for chemical and biological assessments as follows. For the GAC filters, a 50-mL aliquot of the content (slurry and any biofilms it contained) was collected and filtered. The filtrate was acidified with nitric acid and stored for analysis by inductively coupled plasma-mass spectrometry (ICP-MS). The remaining aliquot was apportioned into two parts. One part was saved in refrigerator at −80 °C for biological analysis and remaining portion was dried at 60 °C and saved for the leaching process. For polypropylene filters, the murky water in the cartridge chamber was collected and filtered through 0.45 *μ*m membrane. The filtrate was acidified with nitric acid and preserved for trace metal/metalloid analysis. The residues on the cartridge were carefully washed off with deionized water into a beaker (strictly no scraping to avoid filter material contamination). The sediment was filtered through 0.45 *μ*m membrane and the cake dried at 60 °C and saved for leaching process.

### Major ions in water samples by RF-HP-IC

Major inorganic anions (F, Cl, Br, NO_3_, PO_4_, and SO_4_) and cations (Na, NH_4_, K, Mg, and Ca) in un-acidified samples were determined on Dionex (Sunnyvale, CA) high performance ion chromatography system (RF-HP-IC) located at the QEERI Laboratory in Doha following the Standard Methods of APHA^49^. A 25-µL of sample was injected from the auto sampler at a flow rate of 1 mL/min and 30 °C temperature for both anions and cations. The anions were separated on IonPac® AS19 (Dionex, Sunnyvale, CA) analytical anion-exchange column and IonPac® AG19 guard column using a gradient KOH eluent electronically generated in line. The elution was then carried out with 10 mM at gradient of KOH from 0 to 10 minutes and 58 mM from 10 to 40 minutes. The cations were separated on IonPac® CS12A analytical cation exchange column with IonPac® CG12A guard column using electrolytically generated methanesulfonic acid (MSA) eluent. Cations were eluted isocratically with 20 mM MSA. In the beginning of each batch of analysis, a calibration curve of working standards was prepared and a regression analysis was performed for each analyte (anions and cations). Working standards were prepared from stock anions solution [Dionex Seven Anion Standard II containing F (20 mg/L), Cl (100 mg/L), NO_2_ (100 mg/L), Br (100 mg/L), NO_3_ (100 mg/L), PO_4_ (200 mg/L), SO_4_ (100 mg/L)] and cation solution [Dionex Six cation-II Standard] containing Li (60 mg/L), Na (200 mg/L), NH_4_ (250 mg/L), K (500 mg/L), Mg (250 mg/L), Ca (500 mg/L)].

Data quality objectives were achieved by implementing stringent quality assurance and quality control (QA/QC) protocols. Freshly prepared working standards in linear calibration ranges were used for each batch of samples. Field and laboratory blanks were analyzed with each batch of sample to assess any change, contamination or instrumental drift. QC check, spiked and duplicates were scheduled within each batch to check the precision and reproducibility. Field blanks were run at the start and end of each batch of analysis in addition to duplicate, laboratory reagent blank and the calibration blank that were analyzed after every ten samples. Data validation for a batch was acceptable if laboratory control sample (QC check) and the continuing calibration verification solution recovery fell within the limits of 90–110%. Matrix spike was run after fifteen samples or once in the batch and the acceptance criteria was achieved if recovery of the spiked analyte was in the 80–120% range. Calculations were automatically performed by the Chromeleon® software (Dionex) integrated with ICS-5000 system and results were reported in mg/L.

### Trace elements in water samples by ICP-MS

Water samples acidified in the field were filtered through 0.45 µm membrane. The US EPA Method 200.8^50^ was followed for multi-element determination of trace elements in water samples using an ICP-MS located at the QEERI Laboratory in Doha. A Bruker Aurora Elite (Bruker, Bremen, Germany) ICP-MS instrument was used in this investigation. The instrument was operated in normal sensitivity mode with 30 scans/replicates in peak hopping scan mode and RF power 1.3 kW. A blank solution of 1% v/v HNO_3_ (Ultrapure, Merck) and pure deionize water (18MΩ cm) were used to obtain the instrument detection limits i.e. three times the standard deviation of 10 replicates of the blank.

External calibration standards were prepared with Bruker Daltonics Calibration mix2 ICP-MS multi-element stock solution. Strict quality assurance (QA) and quality control (QC) procedures were employed to achieve the data quality objectives. Assessment of the level of accuracy and quality control included NIST (National Institute of Standards and Technology, Gaithersburg, MD) certified reference material 1640 (trace elements in natural water) measurements, determination of spike recovery for selected heavy metals, and measurement of duplicates for each batch of ten samples. Achieved recoveries ranged between 87% and 105% with percent relative standard deviation (%RSD) for the mean recoveries being below 8.7%. The matrix spike recoveries ranged from 82% to 112% with %RSD below 10%. The %RSD for duplicate measurements was less than 7% in all batches. Internal standard from Bruker Daltonics (Mix of Li6, Sc, Y, In and Tb in 2% HNO_3_) was added to reduce matrix interference and check for instrumental drift.

### Suspended solids in filter cartridge

Filter residues were leached using 0.5 M nitric acid instead of harsh acid digestion that might complicate the results by mineralizing the activated carbon or polypropylene filter material. A known amount (~0.5 gram) of the dried residue (from GAC or PP filters) were mixed with 5 ml of 0.5 M ultrapure nitric acid (Merck) in a beaker and left at room temperature for 24 hours. The mixture following room-temp digestion was filtered through 0.45 µm membrane and diluted to 10 ml with deionized water and saved for ICP-MS analysis as described above.

### Total organic carbon (TOC)

Water samples collected in 40 mL amber glass bottles were preserved with sulfuric acid. They were subsequently analyzed using Shimadzu TOC-L (Shimadzu, Kyoto, Japan) total organic carbon analyzer within two weeks after collection.

### Microbiological assay

Samples were collected for microbiological characterization using sterilized glass containers and then kept on ice until processed in the laboratory. Samples were held for 4–10 hours before being processed in the lab. Standard microbiological assay was used to determine the heterotrophic colony forming units in each sample^51^. Although the development of heterotrophic plate count (HPC) or colony forming units (CFU) is no longer considered a significant health risk per se (culture temperature and medium can influence the results, for instance), it is still valued as an indicator of water quality and treatment efficacy^51,52^. We used this standard and traditional technique for microbiological testing in this study because the method continues to figure in water regulations and guidelines in most countries as an indicator of the general microbiological quality^3,32,53^.

Briefly for the heterotrophic plate counts (HPC), 100 mL of the sample was passed through 47 mm diameter, 0.45 *µ*m cellulose nitrate filters and transferred to R2A agar growth medium. The 100-*µ*L and 1 mL of water sample in duplicate were directly spread onto R2A agar plates and incubated at 30 °C for three days. Plates were counted and morphology was noted at the end of the incubation. The numbers of HPC was normalized by volume to get standardized counts per 1.0 mL. The highest reported count was 490/plate (100 *µ*L) and any value of 550/100 *µ*L or higher was assigned to too many to count (TMTC) category.

### DNA genotyping

The approach used for identification of main microbial strains was cultivation of microbes on R2A, followed by amplification and partial sequencing of 16S rRNA genes (Eichler *et al*.^10^). Amplification of the hypervariable regions 2, 4 and 8 and regions 3, 6–7 and 9 of the 16S bacterial rRNA gene was performed on all samples using Ion 16S^TM^ Metagenomics Kit (Thermo Scientific, Waltham, MA). 1–3 ng of the PCR amplification products were used to create a library using the Ion Plus Fragment Library Kit (Thermo Scientific, Waltham, MA) with specific barcodes used to label each fragment based on sample number (Ion Xpress Barcode Adaptors; Thermo Scientific, Waltham, MA). PCR amplicons were purified using Agencourt AMPure XP (Beckman-Coulter Inc, Brea, CA) Bead-based size selection. The Agilent® High Sensitivity DNA Kit was used to quantify DNA concentration for input for library preparation. Each library was diluted to 26 pM for template preparation, which was performed using the IonChef System and the Ion 520™ & Ion 530™ Kit-Chef (Thermo Scientific, Waltham, MA). Sequencing was conducted on the Ion S5 system using the 330 ^TM^ chips.

The 16S rRNA workflow module in the Ion Reporter TM software was used to classify individual reads via a two-step alignment to the manually curated MicroSEQ® and public content Greengenes databases. In the first step, reads are aligned to the MicroSEQ® ID library with any unaligned reads subject to a second alignment to the Greengenes database^54,55^. Taxonomical assignments in this data set are reported as a consensus of the results from all of the primers. By default, alignment at various taxonomic levels follows the Clinical and Laboratory Standards Institute (CLSI) guidelines requiring the family level to have <97% identity, with genus >97% identity and species >99% identity.

### Data analysis

The survey results were analyzed using SPSS Statistics 24 (IBM Inc., Armonk, NY). Descriptive statistics were first calculated. The Pearson moment was used to evaluate the linear relationships between various pairs of variables, with statistical significance set at *p* < 0.05. Linear multivariate models (LMM) were employed to explore the relationships between HPC, TOC and TC (outcomes of particular interest as multiple dependent variables) and the various measures of water quality in the study. LMM models were also used to assess the interactions between the three outcomes of interest. The value of correlation coefficient in these regression models can range between −1.0 (a perfect inverse relationship between the two variables) and +1.0 (the two variables are co-linear and react in exactly the same way as their values change). A correlation coefficient of zero suggests that the two variables are independent of each other.

### Disclaimer

The views expressed here are those of the authors and do not necessarily represent the views or policies of any of the institutions where they work.

### Data availability statement

The datasets generated during and/or analyzed during the current study are available from the corresponding author on reasonable request.

## Electronic supplementary material

Dataset 1
