# Supplementary material for: Influence of Household Water Filters on Bacteria Growth and Trace Metals in Tap Water of Doha, Qatar
Source: Sci Rep. 2018 May 29;8:8268. doi: 10.1038/s41598-018-26529-8 (PMC5974232; doi:10.1038/s41598-018-26529-8)
Supplement: Supplementary file 1 — Dataset 1 [file 41598_2018_26529_MOESM1_ESM.docx]

**Influence of Household Water Filters on Bacteria Growth and Trace Metals in Tap Water of Doha, Qatar**

*Nriagu J, Xi C, Siddique A, Vincent A, Shomar B.*

**SUPPLEMENTARY DATA**

Suppl 1. Geographic coordinates of households used in the study

| **System No** | **Location** | **Coordinates** | |
| --- | --- | --- | --- |
|  |  | **Latitude** | **Longitude** |
| **1** | Ezdan Mall | 25.330845 | 51.453805 |
| **2** | Al-Wadi Compound | 25.349791 | 51.442099 |
| **3** | Fareej Abdulaziz | 25.277639 | 51.521668 |
| **4** | Al- Muntazah | 25.270732 | 51.523537 |
| **5** | Nauija | 25.244172 | 51.538318 |
| **6** | Bin Mahmood | 25.286975 | 51.511702 |
| **7** | Muntazah | 25.270783 | 51.523771 |
| **8** | Lavendar compound | 25.346452 | 51.449188 |
| **9** | Lavendar compound | 25.346713 | 51.448608 |
| **10** | Al- Ghanim area | 25.346713 | 51.448608 |
| **11** | Ain Khalid | 25.217688 | 51.438095 |
| **12** | Naujia | 25.249007 | 51.527227 |
| **13** | Duhail | 25.35569 | 51.475252 |
| **14** | Musherib | 25.282692 | 51.530547 |
| **15** | Bin Omran | 25.300197 | 51.498745 |
| **16** | Industrial area | 25.196379 | 51.431737 |
| **17** | Ain Khalid | 25.224474 | 51.457857 |
| **18** | New industrial area | 25.218703 | 51.434403 |
| **19** | Abu Hamour | 25.237084 | 51.489933 |
| **20** | Ar Rehan | 25.291797 | 51.416529 |
| **21** | Old Indutrial Area | 25.165254 | 51.432815 |
| **22** | Muaither | 25.268904 | 51.419036 |
| **23** | Education city | 25.306024 | 51.445387 |
| **24** | Khartiyat Café | 25.38093 | 51.436156 |
| **25** | Al- Thumama | 25.237089 | 51.56996 |
| **26** | Madina Khalifa | 25.319844 | 51.482543 |
| **27** | Al- Hatimi | 25.284498 | 51.545432 |
| **28** | Najma | 25.268181 | 51.536863 |
| **29** | Al-Salata | 25.285788 | 51.547027 |
| **30** | Al-Soudan | 25.266786 | 51.487261 |
| **31** | Al Sailiya | 25.209836 | 51.376738 |
| **32** | New Sailiya (Abu Nakhla) | 25.184495 | 51.384143 |
| **33** | Muntaza | 25.270034 | 51.523593 |
